# Single-cell transcriptome and cell-specific network analysis reveal the reparative effect of neurotrophin-4 in preantral follicles grown in vitro

**DOI:** 10.1186/s12958-021-00818-w

**Published:** 2021-09-04

**Authors:** Yingchun Guo, Peigen Chen, Tingting Li, Lei Jia, Yi Zhou, Jiana Huang, Xiaoyan Liang, Chuanchuan Zhou, Cong Fang

**Affiliations:** Reproductive Medicine Research Center, Sixth Affiliated Hospital of Sun Yat-Sen University, Guangzhou, 510275 Guangdong China

**Keywords:** In-vitro-grow, Preantral follicle, Single-cell transcriptome, NT-4, Fertility preservation

## Abstract

**Background:**

In-vitro-grow (IVG) of preantral follicles is essential for female fertility preservation, while practical approach for improvement is far from being explored. Studies have indicated that neurotrophin-4 (NT-4) is preferentially expressed in human preantral follicles and may be crucial to preantral follicle growth.

**Methods:**

We observed the location and expression of Tropomyosin-related kinase B (TRKB) in human and mouse ovaries with immunofluorescence and Western blot, and the relation between oocyte maturation and NT-4 level in follicular fluid (FF). Mice model was applied to investigate the effect of NT-4 on preantral follicle IVG. Single-cell RNA sequencing of oocyte combined with cell-specific network analysis was conducted to uncover the underlying mechanism of effect.

**Results:**

We reported the dynamic location of TRKB in human and mouse ovaries, and a positive relationship between human oocyte maturation and NT-4 level in FF. Improving effect of NT-4 was observed on mice preantral follicle IVG, including follicle development and oocyte maturation. Transcriptome analysis showed that the reparative effect of NT-4 on oocyte maturation might be mediated by regulation of PI3K-Akt signaling and subsequent organization of F-actin. Suppression of advanced stimulated complement system in granulosa cells might contribute to the improvement. Cell-specific network analysis revealed NT-4 may recover the inflammation damage induced by abnormal lipid metabolism in IVG.

**Conclusions:**

Our data suggest that NT-4 is involved in ovarian physiology and may improve the efficiency of preantral follicle IVG for fertility preservation.

## Background

In vitro follicle culture is an essential issue for female fertility preservation (FP) and research of folliculogenesis. Since females confronted with potentially gonadotoxic treatment have a greater opportunity for long-term survival than ever before, there is a general consensus on providing FP options [1, 2]. However, due to limited time or contraindication of ovarian stimulation, the harvest of mature oocytes from antral follicles is far less than the number required for a take-home baby [3, 4]. By comparison, preantral follicles are considered a richer oocyte banking than antral follicles and more achievable than activation of primordial follicle. For patients with the risk of reseeding cancer cells when transplanting cryopreserved tissue, in-vitro-grow (IVG) of preantral follicles could eliminate the risk [5]. Investigations on isolated follicle IVG have been performed for mice [6–8], domestic animals [9, 10], non-human primates [11–14], and humans [5, 15–17]. The recent encouraging study reported the production of metaphase II human oocytes [18] following IVG. However, only nine oocytes with abnormal polar bodies were achieved with 136 pieces of ovarian tissue. Moreover, the comparison between oocytes derived from extended IVG versus in vivo was not fully investigated. Considerable researches are needed to confirm the efficacy and safety of oocytes derived from preantral follicle IVG before used in fertility preservation.

Follicular growth in vivo involves a series of precise intraovarian and neuroendocrine regulators. Microenvironment of the existed IVG systems simulate only a small portion of neuroendocrine regulators, which intraovarian factors could not compensate. Accumulating evidence has suggested neurotrophins’ roles in follicle assembly, follicular growth and oocyte maturation [19, 20]. Neurotrophin-4 (NT-4), a member of neurotrophins, has been identified as distinct stage-specific and preferentially expressed in human preantral and antral follicles [21]. With gene knock-out mice, study indicated that the deficiency of TRKB, the tyrosine kinase receptor for NT-4, decreased secondary follicles and FSH receptor expression in the ovary [22]. However, the role of NT-4 in the IVG of mammalian preantral follicles is far from being explored.

In this study, we observed the dynamic location of TRKB in ovaries of human and mouse, and explored the relation between oocyte maturation and level of NT-4 in the follicular fluid (FF) aspirated from follicles of in vitro fertilization (IVF) or Intracytoplasmic sperm injection (ICSI) cycles. Then we first investigated the effect of NT-4 on preantral follicle IVG applying mice model, to develop a more effective and safe method for female fertility preservation. Single-cell RNA sequencing of oocyte combined with cell-specific network analysis was conducted to uncover the underlying mechanism of effect, which is also the first high-throughput data comparing oocyte derived from preantral follicle IVG versus in vivo.

## Methods

### TRKB receptor identification in human and mouse ovaries

#### Source and collection of ovaries

This study was approved by the ethics committee of the Reproductive Medicine Centre of the Sixth Affiliated Hospital of Sun Yat-sen University (2014ZSLYEC-002S), and all participants had signed the informed consent on enrollment. The ovarian specimens used for immunohistochemical studies derived from 5 women diagnosed with nongynecological cancer from 26 to 33 years old, undergoing pelvic surgery for cancer treatment or ovary cryopreservation. Mouse ovaries were collected from female Kunming mouse aged 2-week-old and 6-week-old. Cumulus oocyte complexes (COCs) growing in vivo were collected by antral follicle puncture. Oocytes and granulosa cells were denuded by gently pipetting in a droplet of PBS buffered medium containing 80 IU/ml hyaluronidase (Vitrolife, Sweden). Oocytes, somatic granulosa cells and cumulus cells (CCs) were collected respectively and washed twice with phosphate-buffered saline (PBS), then stored at -80 °C until Western blot or RNA extraction. All the animal procedures were performed under the ethical guidelines of the Laboratory Animal Center of Sun Yat-sen University.

#### Immunofluorescence

Formalin-fixed, paraffin-embedded ovarian specimens were sectioned at five μm. After dewaxing, antigens were retrieved by pressure cooking in sodium citrate buffer at 90 °C for 40 min. Sections were blocked for 30 min in 3% diluted in phosphate-buffered saline (PBS; pH7.4). The immunoreactions were performed with the TRKB (Novus biologicals, Bio-Techne, USA) antibody (10 μg/ml) and DDX4 (Abcam, USA) antibody (1:100) simultaneously. Slides incubated in the absence of primary antibodies were used as a negative control. After an overnight incubation at 4 °C, the primary antibody was detected using fluorochrome-tagged secondary antibodies by incubating the sections with Alexa Fluor 488 Goat Anti-Mouse (Abcam, USA; 1:400) and Cy™3 AffiniPure Donkey Anti-Goat IgG (Jackson ImmunoResearch Laboratories, Inc., West Grove, PA; 1:400) for 1 h at room temperature. Cell nuclei were stained with DAPI dihydrochloride (Solarbio Life Sciences, China). Confocal images were acquired using a Leica Corp. TCS SP8 confocal system (Leica Corp. Microsystems, Heidelberg, Germany). In general, six to eight representative sections were acquired for each image. Images were further processed using Photoshop.

#### Western blot

Ovaries or denuded granulosa cells were stored at -80 °C. Samples were lysed using Sodium dodecyl sulfate (SDS) sample buffer, boiled for 5 min, separated on SDS-PAGE gel, and then transferred onto a polyvinylidene difluoride (PVDF) membrane. The membrane was blocked in 5% low-fat milk for 1 h and incubated at 4 °C with 1:400 goat anti-TRKB polyclonal antibody (Novus biologicals, Bio-Techne, USA) overnight. Membranes were washed three times using PBST buffer, and then incubated with anti-goat rabbit-radish peroxidase-conjugated secondary antibody (1:5000) for 1 h at room temperature. Finally, membranes were processed using a chemiluminescence detection system. Detected bands of predicted size for TRKB and GAPHD (an internal control for normalization) were quantified using ImageJ software.

### Human samplea collection and NT-4 assay

#### Participants

Forty-nine patients undergoing IVF/ICSI in the Reproductive Medicine Center of the Sixth Affiliated Hospital of Sun Yat-sen University were included, from September to October 2020. The inclusion criteria were: (1) female age < 40 years, (2) underwent IVF or ICSI treatments, (3) the number of oocytes retrieved > 3 and AMH (anti-Müllerian hormone) ≥ 1·1 ng/ml, (4) regular menstrual cycles over the previous 3-month period. Couples were excluded if female patients were diagnosed with adenomyosis, uterine cavity abnormalities, untreated hydrosalpinx, immunologic disease, or had any contraindications to progesterone and Gn (gonadotrophin) use. Male patients whose sperm was collected by surgery were not included in the study. Cycles administrating in vitro maturation or preimplantation genetic treatment were excluded. This study was approved by the ethics committee of the Sixth affiliated hospital of Sun Yat-sen University (2020ZSLYEC-277) and all participants had signed the informed consent on enrollment.

#### Follicular fluid retrieval procedures and Cumulus cells collection

During IVF/ICSI treatment, patients underwent controlled ovarian hyperstimulation (COH) and follicle growth was monitored by transvaginal ultrasonography every 2 to 4 days. When at least one follicle reached a diameter of 18 mm, or two follicles reached 17 mm, recombinant hCG (Ovitrelle, Merck-Serono, Italy) 250 μg or hCG (Lizhu Pharmaceutical Trading Co., China) 10,000 IU were administrated. Oocyte retrieval was performed 36 h later. FF aspirates were centrifuged at 2000 rpm for 10 min to remove cellular components and debris. Supernatants were collected for storage at -80 °C until assay. FF NT-4 was assessed using enzyme-linked immunosorbent assay kits (Elabscience, China) according to the manufacturer’s instructions, with a range of 31·25 to 2000 pg/ml and detection sensitivity of 18·75 pg/ml. Intra- and inter-assay coefficients of variation were less than 10%. COCs were collected and incubated at 37 °C and 5% CO_2_ for at least 2 h. Then we denuded CCs by gently pipetting in a droplet of HEPES buffered medium containing 80 IU/ml hyaluronidase (Vitrolife, Sweden). CCs of each patient were collected and washed twice with PBS, then stored at -80 °C until Western blot detection. Fertilization, embryo culture, and transfer were carried out following standardized procedures.

#### Grouping and Statistical analysis

The clinical outcomes were recorded, including the number of mature oocytes, normal fertilization embryos, available embryos, high-quality embryos, and blastocysts. For analysis, cases were grouped according to the rate of mature oocytes with a threshold value of 80%. NT-4 levels in FF, patient demographics, and stimulation characteristics were compared between groups with two-tailed Student’s t-tests, and *P* < 0·05 were considered as statistically significant. Binary logistic regression analysis was conducted to assess the potential factors related to the rate of oocyte maturation. The dependent variable was defined as followed: the rate of oocyte maturation less than 80% is defined as ‘0’; the rate of oocyte maturation higher than 80% is defined as ‘1’.

### In vitroculture of mouse preantral follicle

#### Animal, follicle dissection and In vitro culture

Animals were housed under controlled temperature (25 °C) and light-controlled conditions (12 h light/day). Female Kunming mice aged 12 to 14 days were sacrificed by cervical dislocation, and the ovaries were collected. Follicle dissection was performed according to the procedure adapted from the Woodruff and Shea Labs protocols [23] (Fig. 2a). In brief, ovaries were obtained and transferred in a 4 °C dissection medium, which contains L15 medium (Leibovitz, Gibco) supplemented with 10% of fetal bovine serum, 100 U/ml of penicillin, and 100 mg/ml of streptomycin (Biotopped Life Sciences, China). Individual preantral follicles were mechanically dissected by two 29G^1/2^ insulin syringes gently in prewarm dissection medium on 37 °C heated stages. Follicles measuring 110 ± 20 μm in diameter with a centrally placed oocyte and no signs of somatic cell degeneration were picked for further culture. Individual follicles were placed in 96-V-well microtiter plates in 100-μL preequilibrated (5% CO_2_) and prewarmed (37 °C) culture medium: α-MEM (Gibco, US) supplemented with 1 mg/mL of fetuin (Sigma, US), 1% of ITS (insulin 10 mg/L; transferrin 5·5 mg/L; sodium 5 μg/L) (Sigma, US), 3 mg/mL of albumin bovine (Sigma, US) and 10mIU/mL of FSH (ProSpec, Israel). To determine the optimal dose, different concentrations of NT-4 and medium alone were administered. Cultures were carried out in the incubator with or without NT-4 (0, 50, 100, 200 ng/ml) for ten days at 37 °C and 5% CO_2_ in air (Application number of national invention patent of China: 202,110,274,259.5). The refreshment was performed every other day by replacing half of culture medium and the corresponding NT-4 supplement, and spent medium was collected and stored at -80 °C until assay. Experiments for all groups were conducted in parallel, and 388 follicles from 3 replicated experiments were analyzed.

#### Evaluation of follicle survival and hormone Assay

The diameter of follicles was assessed every other day. Follicles were considered atresia or degenerated if the oocyte became dark or disappeared or the growth of follicle diameter became stagnant. Antrum formation was determined by the presence of a visible translucent area in the follicle at the end of the culture. Steroid hormones were measured by enzyme-linked immunoassay kit (lowest amount detectable for estradiol-17 β and progesterone were 5 pg/mL and 0·03 ng/mL, respectively; Cusabio Biotech, China).

#### In vitro maturation

Cumulus oocyte complexes (COCs) were released from antral follicles at the end of IVG and incubated in preequilibrated and prewarmed mature medium containing α-MEM supplemented with 1.5 IU/mL hCG (Ningbo Second Hormone Factory, China) and 10% FBS (Gibco, US) at 37 °C and 5% CO_2_ for 12 to 16 h. Then oocytes were released from the surrounding cumulus cells by gently pipetting in a droplet of HEPES containing 80 IU/ml hyaluronidase (Vitrolife, Sweden). The appearance of the first polar body served as markers for complete oocyte nuclear maturation.

#### Oocyte and granulosa cells (GC) collection

For comparison, COCs growing in vivo were collected by antral follicle puncture. Oocytes in the germinal vesicle stage growing in vivo or in vitro (Day 6 and Day 10) were released from COC as previously stated. Each sample containing six oocytes was collected in lysate buffer for SMARTer cDNA synthesis and library construction. Each group contained three oocyte samples from three replicated experiments. The remaining cumulus granulosa cells were collected and kept at -80 °C until the assessment of gene expression.

### Single cell RNA sequencing analyses of mouse oocyte

#### SMARTer cDNA synthesis and library construction

Whole oocyte total RNA was amplified using the SMARTSeq2 protocol. Briefly, Oocytes were lysed, and first-strand cDNA synthesis was conducted with a modified oligo(dT) primer (the SMART CDS Primer). The resulting full-length, single-stranded (ss) cDNA was amplified by LD PCR to get enough dscDNA, and cDNA was fragmented by dsDNA Fragmentase (NEB, M0348S). For library construction, blunt-end DNA fragments were generated, and size selection was performed. A-base and indexed Y adapters were administrated to obtain ligated products. After amplifying ligated products with PCR, paired-end sequencing was performed with an Illumina Novaseq™ 6000 (LC Sciences, USA).

#### Primary analysis and RNA-seq reads mapping

Reads containing sequencing adaptors or sequencing primer or nucleotide with q quality score lower than 20 were considered low quality and removed. Using the HISAT2 package [24], the remaining reads were aligned to the mus musculus reference genome mm10 from the University of California Santa Cruz (UCSC, http://genome.ucsc.edu). The mapped reads of each sample were assembled and merged to reconstruct a comprehensive transcriptome using StringTie [25]. Then, the expression level for mRNAs was assessed by calculating FPKM with StringTie.

#### Heatmap, Principal Component Analysis (PCA) and differentially expressed genes analysis

The RNA-seq normalized data were subjected to heatmap analysis and PCA, using Seurat method and only highly variable genes (coefficient of variation > 0·5) were used as inputs. Single-cell data of 9 GV oocytes in IVV, IVG, and IVG-NT groups were analyzed to observe the whole clustering profile. The selection of differentially expressed genes (DEGs) were performed by using R package edgeR (https://bioconductor.org/packages/release/bioc/html/edgeR.html) with the log2 |fold change|> 1 and *P* < 0·05.

#### Functional enrichment analysis

Gene Ontology (GO), Kyoto Encyclopedia of Genes and Genomes (KEGG) analysis were conducted for each list of DEGs and PPI subnetworks by using Gene Set Enrichment Analysis (GSEA) [26] and ClusterProfiler [27] with adjusted *P* < 0·05 as the cutoff.

#### Transcription Factor prediction and Protein–protein network (PPI) construction

We used a reference TF-target interaction database -TRRUST v2 (Transcriptional Regulatory Relationships Unraveled by Sentence-based Text mining version 2 [28] to predict the major factors in transcriptional regulation. The PPI network was constructed by the Search Tool for the Retrieval of Interacting Genes (STRING) database [29] and visualized by Cytoscape (version 3.6.1) software [30].

### Bulk RNA-seq analysis of mouse granulosa cell

#### mRNA library construction and sequencing

Total RNA of each sample was extracted and purified using TRIzol reagent (Invitrogen, Carlsbad, CA, USA), followed by fragmentation using Magnesium RNA Fragmentation Module (NEB, USA), and synthesis of U-labeled second-stranded DNAs using SuperScript™ II Reverse Transcriptase (Invitrogen, USA), E. coli DNA polymerase I (NEB, USA), RNase H (NEB, USA) and dUTP Solution (Thermo Fisher, USA). After single- or dual-index adapters were ligated to the fragments, the U-labeled second-stranded DNAs were treated with heat-labile UDG enzyme (NEB, USA), and the ligated products were amplified with PCR. Sequencing data were generated on Illumina Novaseq™ 6000 (LC Sciences, USA) with 2 × 150 bp paired-end sequencing (PE150) following the vendor's recommended manual.

#### Sequence and DEGs analysis

Raw RNA-seq reads that contained adaptor contamination or with low-quality bases were discarded using Cutadapt software (https://cutadapt.readthedocs.io/en/stable/, version:cutadapt-1.9). Clean reads were mapped against the genome (mus musculus Ensembl v99) using HISAT2 [24] software. The mapped reads were assembled administrating StringTie [25] with default parameters. After all transcriptomes were generated, the expression levels were estimated with StringTie and ballgown (http://www.bioconductor.org/packages/release/bioc/html/ballgown.html). Gene expression levels were quantified with FPKM. DEGs were selected with fold change > 2 or < 0·5 and *P* < 0·05 by R package edgeR, and Benjamini & Hochberg FDR was calculated for statistical significance of DEGs. GO enrichment and KEGG enrichment of the DEGs were analyzed with R package ClusterProfiler [27] and GSEA [26].

### Cell communication inference

We used CellTalkDB which contains literature-supported ligand-receptor (LR) pairs for human and mouse to identify cross-talk signals from oocyte and GC cell RNA-seq data based on database curated from PPIs in STRING database and verified manually in Pubmed by three reviewers [31].

### Quantitative reverse transcription PCR (qRT-PCR) validation

#### Single cell full length mRNA amplification and DNA libraries construction

The oocytes and GCs that were isolated from follicles were lysed and the total RNAs were amplificated and purified using a Single Cell Full Length mRNA-Amplification Kit (Vazyme, N712) according to the manufacturer’s instructions. Briefly, the first-strand cDNA was synthesized from the whole lysate using the Oligo primer containing a 24-nt poly(dT) tail at the 3’ end. Then, the first-strand cDNA was amplified by PCR using PCR primer. Amplified cDNA was purified with VAHTS DNA Clean Beads and evaluated by Agilent 2100 Bioanalyzer (Agilent Technologies). The prepared cDNAs were sheared into fragments, and used for preparation of deep sequencing libraries. The DNA-Seq libraries were constructed by a TruePrep DNA Library Prep Kit V2 (Vazyme, TD502-02).

#### Real-time Quantitative Reverse Transcription Polymerase Chain Reaction

Quantitative reverse transcription PCR (qRT-PCR) analyses were performed on a LightCycler 480 II system (Roche, Switzerland). The reaction solution (10 μL) consisted of 1μL of cDNA, 5μL of 2xRealStar Green Power Mixture (GenStar), 0.5 μL of primer mix, and 3.5 μL of ddH_2_O. Thermal cycling parameters were set up as follows: 95 °C for 10 min, followed by 50 cycles at 95 °C for 15 s and 60 °C for 1 min. The 2^(−△△ct)^ method was applied to calculate the relative expression of targeted genes. Transcripts were quantified in triplicate experiments, and Actin was used as the reference gene. The primer sequences used are presented in Table 1.

**Table 1:** Primers for qRT-PCR

**Gene (mmu)**	**Forward primer**	**Reverse primer**
*Ntrk2*	TCCAGCACGAGCACATTGTCAAG	TTCTCTCCCACCAGGCAGTTCC
*Cacna1s*	CAGCAGAGGAGGAACTGGAGAGG	ATTGGCATTGGCGTTGTTGGTATTG
*Orail*	AGCAACGTCCACAACCTCAACTC	AGCGGTAGAAGTGAACAGCAAAGAC
*Prkaca*	TTAGACAAGCAGAAGGTGGTGAAGC	CGGTAGATGAGGTCCAGGGAGTG
*Trp53*	TGAACCGCCGACCTATCCTTACC	CTAGGCTGGAGGCTGGAGTGAG
*Rac1*	AACCTGCCTGCTCATCAGTTACAC	TGTCGCACTTCAGGATACCACTTTG
*Arf6*	TTCGGGAACAAGGAAATGCGGATC	TTGTTGGCGAAGATGAGGATGATGG
*Ugt1a7c*	GAAGCCTATGTCAACGCCTCTGG	ATCATCACCATCGGAACTCCATTGC
*Fga*	ATCACACAGGTAAAGCGGTCACTTC	GGATATGTCTAACTCGGTGGCATCG
*C3*	CACACCGAAGAAGACTGCCTGAC	CTGACTTGATGACCTGCTGGATGG

### Statistical analysis

Enumeration data are presented as n, or the means ± standard deviations and analyzed with Student’s t-test or Mann–Whitney U test for comparison of two groups. Categorical data are presented as n/n and analyzed with the chi-square test, Yates' correction, or Fisher's exact probabilities. Logistic regression analysis was conducted to assess the relationship between the rate of oocyte maturation and FF level of NT-4 to eliminate interference from bias factors. Differences in gene expression detected by RT-qPCR were analyzed using the Wilcoxon matched-pair signed-rank test. All the above statistical analyses were performed with SPSS statistics software, version 23.0. *P* < 0·05 was considered statistically significant. For the plotting of RNA-seq data and the subsequent statistical analyses, R software (The R Foundation, https://www.r-project.org) was administrated. In brief, *P* values were adjusted for multiple comparisons according to Benjamini & Hochberg. And significance for enrichment of families was assessed by Fisher’stest. Graphics were created using GraphPad Prism 8.0, the R core package and gplots, ggplot2, RColorBrewer packages.

## Result

### Detection of TRKB receptor in follicles of human and mouse ovaries

**Fig. 1:**
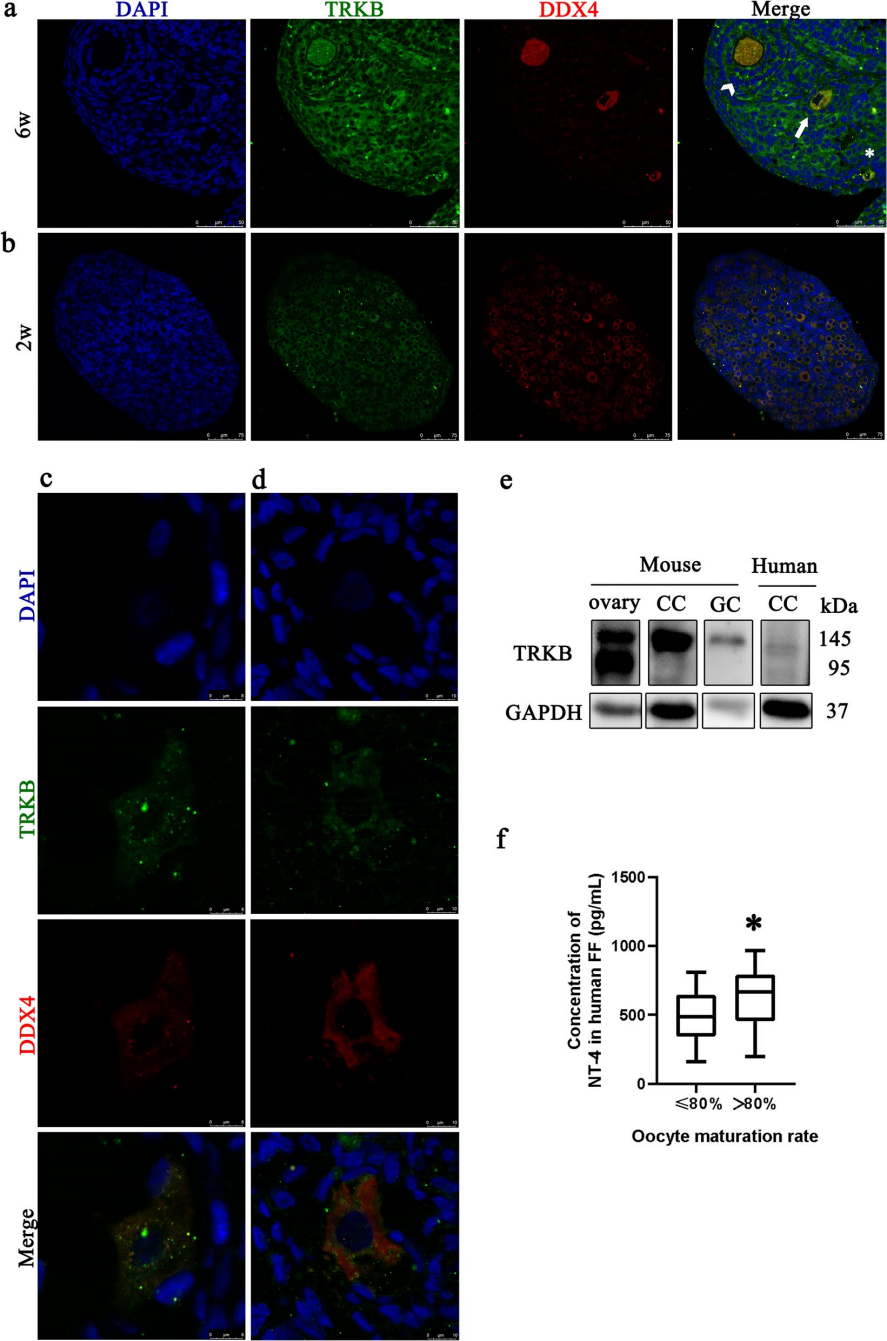
Immunohistochemical localization of TRKB receptor in human and mouse ovaries; human FF level of NT-4. **a** Tropomyosin-related kinase B (TRKB) immunoreactivity is both abundant in GCs and oocyte of 6-week-old mice preantral follicles (arrowhead). In the primary follicle (arrow), the expression of TRKB in GCs is slightly lower than in oocytes. The difference is more significant in primordial follicles (*). **b** TRKB immunoreactivity is present in oocytes of primary follicles and primordial follicles of 2-week-old. GCs contain lower but detectable levels of TRKB protein. **c**, **d** Detection of NGF immunoreactivity in oocytes of human primary or primordial follicles (c) and preantral follicles (d). **a**-**d** These are representative images of three independent experiments. No signal was detected in the negative control for either set of prime. **e** Western blot detection of human cumulus cells, mouse ovary, mouse cumulus cells and mouse somatic granulosa cells of TRKB receptors. **f** FF level of NT-4 from 49 women with different oocyte maturation rates when undergoing IVF/ICSI. *, *P* < 0·05

TRKB immunoreactivity is abundant in oocytes of 6-week-old mice, whereas with the decrease in the size of follicles, the intensity of immunostaining in the GCs decreased (Fig. 1a). In the ovary of 2-week-old mice, TRKB is present in oocytes of primary follicles and primordial follicles. GCs contain lower but detectable levels of TRKB (Fig. 1b). TRKB immunoreactivity is detectable in oocytes of human primary or primordial follicles (Fig. 1c) and preantral follicles (Fig. 1d). With Western blot experiments (Fig. 1e), TRKB was detected in human cumulus cells, mouse ovary, mouse cumulus cells and mouse somatic granulosa cells.

### Correlation between human follicular fluid (FF) NT-4 and oocyte maturation

The mean concentration of NT-4 in FF of 49 women was 532·50 ± 213·70 pg/mL. After patients were grouped according to oocyte maturation rate, comparisons were performed for age, duration of infertility, BMI (body mass index), basal FSH, AMH, antral follicle count, total doses of Gn, and no differences were found. The FF NT-4 level of patients with oocyte a maturation rate of more than 80% is higher than that of the other group (607·76 ± 222·43 vs. 484·84 ± 197·02 pg/mL, *P* < 0·05, Fig. 1f). In order to adjust potential confounders related to oocyte maturation, we conducted a binary logistic regression analysis including age, duration of infertility, BMI, basal FSH, AMH, antral follicle count, total doses of Gn, NT-4 level in FF. The level of NT-4 in FF was positively associated with the probability of a high oocyte maturation rate (more than 80%) (Beta = 0·302, *P* < 0·05) after adjustment of potential confounders.

### Effect of NT-4 on mouse preantral follicle in-vitro-growth

#### NT-4 administration improves the development of preantral follicle and maturation of oocytes

**Fig. 2:**
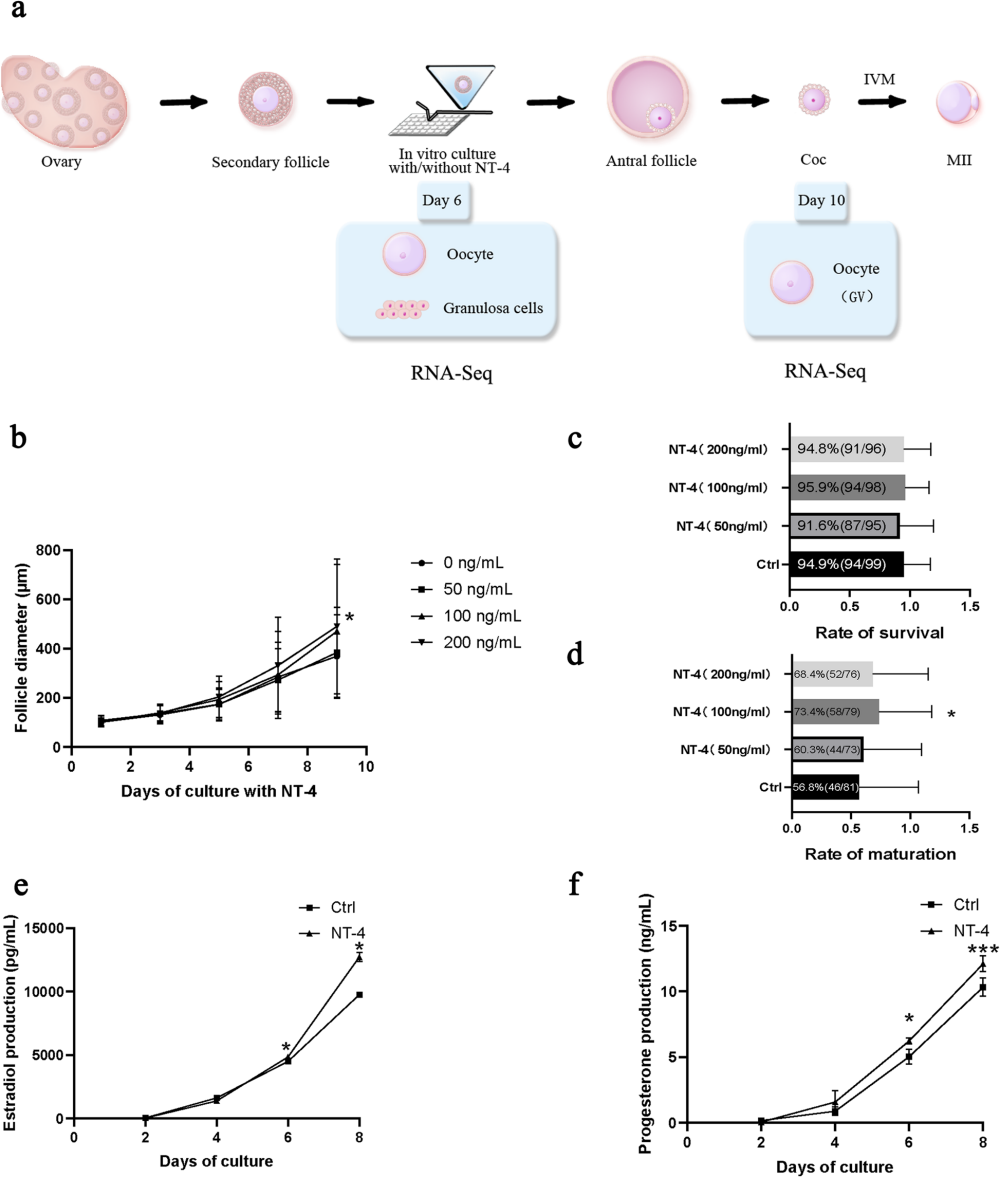
The administration of NT-4 in the culture of mice preantral follicle in vitro. **a** Flow diagram of NT-4 administration to in vitro culture of mice preantral follicle and the corresponding RNA sequencing. Three independent experiments were conducted. **b** Dynamic of follicle diameter when cultured in vitro (0 ng/mL, 50 ng/mL, 100 ng/mL, 200 ng/mL), n = 99, 95, 98, 96, separately. *, *P* < 0·05 0 ng/mL vs. 100 ng/mL and 0 ng/mL vs. 200 ng/mL on Day 9. **c** Survival rate of follicles in Control and NT-4 groups. **d** The rate of Maturation in Control and NT-4 groups. *, *P* < 0·05 Control group vs. NT-4 group (100 ng/mL). **e**, **f** Hormone profiles of estradiol (e) and progesterone (f) in the Control group and NT-4 group (100 ng/mL) during ten days of in-vitro culture. *, *P* < 0·05 Control group vs. NT-4 group. ***, *P* < 0·001 Control group vs. NT-4 group. Data in (b), (e), (f) are presented as mean percentage (mean ± SEM)

The diameter of all follicles increased from 103·49 μm to 428·21 μm on average, and no difference was found on the first day of culture among groups. The addition of NT-4 with concentrations of 100 ng/mL and 200 ng/mL resulted in incremental improvement of follicle growth compared with follicles cultured with medium alone, which reached a statistically significant change on the ninth day of culture (Fig. 2b). At the end of culture, we observed no difference in the survival rate of follicles among groups (Fig. 2c), but a significant increase in the rate of oocyte maturation with the addition of NT-4 at the dose of 100 ng/mL rather than 50 ng/mL or 200 ng/mL (Fig. 2d). We then used a dose of 100 ng/mL NT-4 for our subsequent study.

#### The effect of NT-4 on hormone production of follicles

There were no significant differences in levels of estradiol and progesterone in the culture medium during the first 4 days of culture among groups. Since the sixth day, hormone production was significantly increased in the presence of NT-4, and the difference reached the most obvious on the tenth day of culture (Fig. 2e, f).

### Global RNA analysis of GV oocytes developing in vivo and in vitro

**Fig. 3:**
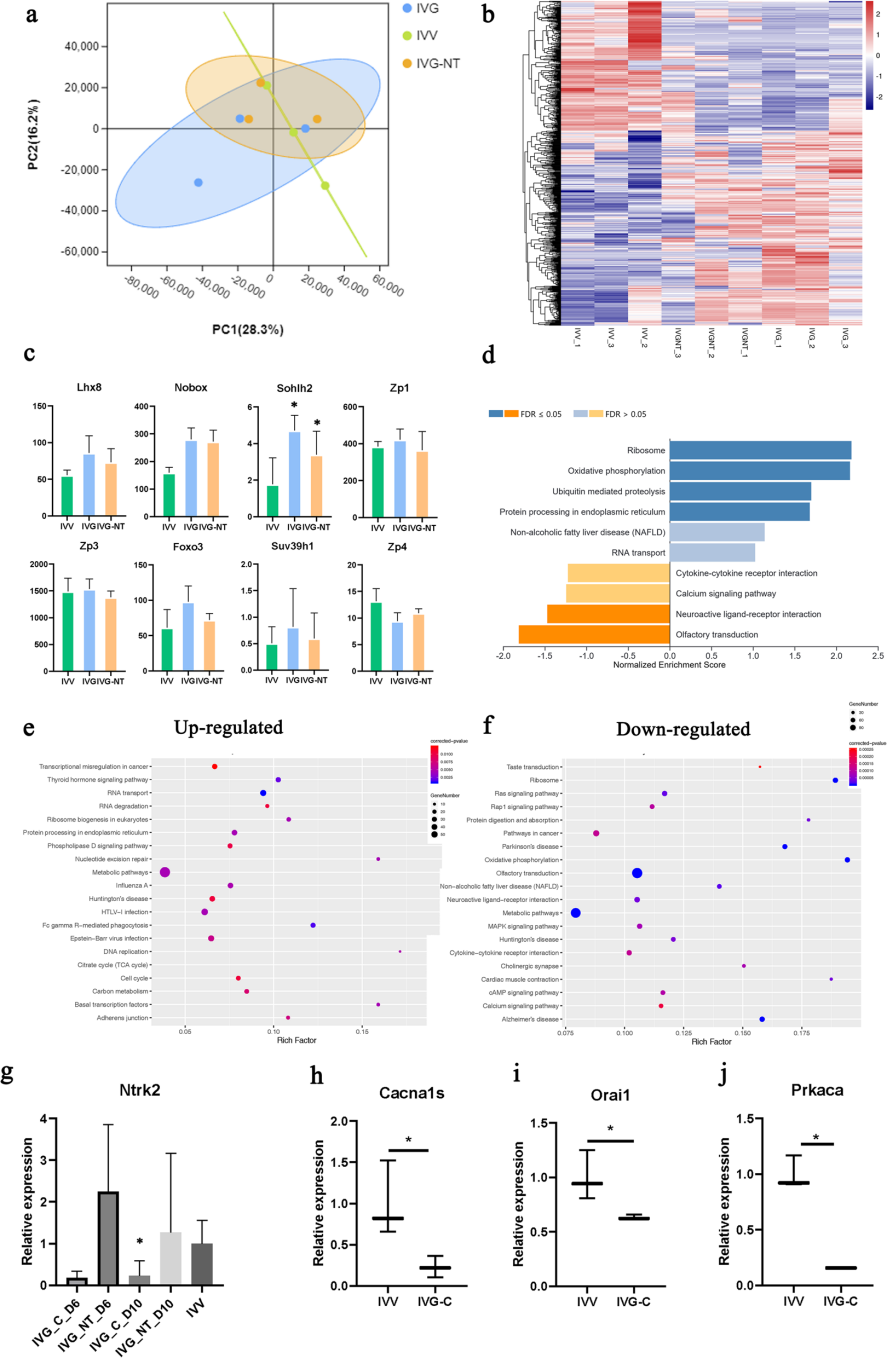
Global RNA analysis of GV oocytes developing in vivo and in vitro. **a** PCA of GV oocytes developing in vivo (IVV), in vitro (IVG), and in vitro with NT-4 (IVG-NT). **b** Gene-expression profiles of genes necessary for oogenesis (*Lhx8*, *Nobox*, *Sohlh2*, *Zp1*, *Zp3*, *Foxo3*) and fully grown oocyte (*Suv39h1*, *Zp4*). The transcription level was calculated by the fragments per kilobase of exon model per million mapped fragments (FPKM). Bars at each point indicate the SD based on three independent experiments. *, *P* < 0·05 IVV vs. IVG, IVV vs. IVG-NT. **c** Heatmap analysis of gene expression profiles of oocytes in IVV, IVG, IVG-NT groups. **d** GSEA enrichment plots of KEGG signaling pathways of upregulated and downregulated different-expressed genes (DEGs) in IVT oocytes compared with IVV oocytes. Blue represents upregulated pathways; orange represents downregulated pathways. **e** KEGG enrichment analysis of upregulated DEGs in IVT oocytes compared with IVV oocytes using DAVID. **f** KEGG enrichment analysis of downregulated DEGs in IVT oocytes compared with IVV oocytes using DAVID. **g** Transcriptional level of TRKB receptor (Ntrk2) gene at different folliculogenesis stages with qRT-PCR experiments. *, *P* < 0·05 IVV vs. IVG of Day 10 oocytes. **h**, **i**, **j** Quantitative RT-PCR detection of *Cacna1s* (h), *Orail* (i), *Prkaca* (j) transcripts in oocytes from Day 10 follicles. *, *P* < 0·05. Each group contains three samples from three independent experiments

Global RNA analysis was conducted with oocytes in the germinal vesicle stage from follicles growing in vivo (IVV) or in vitro for ten days with (IVG-NT) or without NT-4 (IVG). We found that oocytes from these groups had similar percentages of reads mapping in unique or multiple locations. PCA (Fig. 3a) and heatmap (Fig. 3b) revealed a clear separation between IVV oocytes and IVG oocytes, and the IVG-NT samples clustered between IVV and IVG samples.

As a biological control test, we analyzed eight typical genes necessary for oogenesis (*Lhx8*, *Nobox*, *Sohlh2*, *Zp1*, *Zp3*, *Foxo3*) [32] and fully grown oocyte (*Suv39h1*, *Zp4*) [33] (Fig. 3c). Results demonstrated that FPKMs of *Zp3* were similar among groups. The gene expression levels of *Sohlh2* in IVG oocytes were significantly higher than that in IVV oocytes (*P* < 0·05), and IVG-NT oocytes fell in between. A similar pattern was observed in the expression of *Lhx8*, *Nobox*, *Zp1*, *Foxo3* and *Suv39h1* with less significance (*P* > 0·05). On the contrary, *Zp4* was downregulated in IVG oocytes compared with IVV oocytes, and IVG-NT oocytes still fell in between (*P* > 0·05). The transcriptional level of TRKB receptor (Ntrk2) gene at different folliculogenesis stages were validated with qRT-PCR experiments (Fig. 3g). The expression of TRKB receptor in oocytes cultured in vitro without NT-4 was lower than that in oocytes grown in vivo (*P* < 0·05). No significant difference was found between other groups.

For DEGs of oocytes between IVV and IVG groups, KEGG analysis using GSEA (Fig. 3d) showed that downregulated DEGs were highly enriched in neuroactive ligand-receptor interaction, and upregulated DEGs were enriched in ribosome, oxidative phosphorylation, ubiquitin-mediated proteolysis, and protein processing in endoplasmic reticulum pathways (IVG vs. IVV). In addition, KEGG analysis using DAVID revealed that genes enriched in RNA transport, RNA degradation, ribosome biogenesis in eukaryotes, DNA replication, and nucleotide excision repair were upregulated (IVG vs. IVV, Fig. 3e). And genes enriched in neuroactive ligand-receptor interaction, metabolic pathways, MAPK signaling pathway, cytokine-cytokine receptor interaction, cAMP signaling pathway, and calcium signaling pathway were downregulated (Fig. 3f). The transcription of representative genes of cAMP signaling pathway and calcium signaling pathway (*Cacna1s, Orail, Prkaca*) were downregulated in oocytes of IVG group (Fig. 3h, i and j).

### Single-cell transcriptome analysis identifies the effect of NT-4 in GV oocytes

**Fig. 4:**
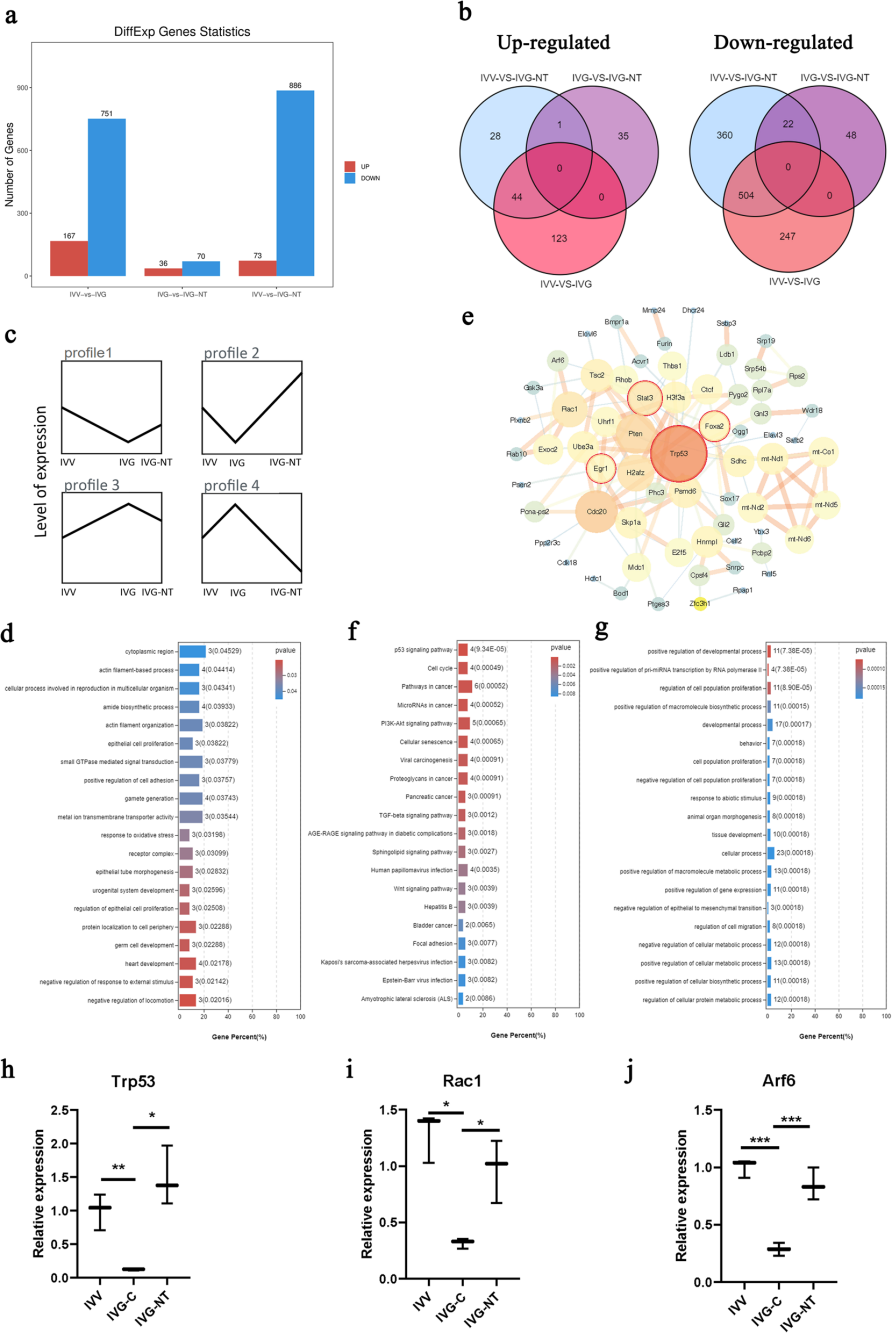
Effect of NT-4 supplementation on transcriptome profiling of GV oocytes. **a** Number of different-expressed genes (DEGs) of each two groups among IVV, IVG, and IVG-NT group. Each group contains three samples from three independent experiments. **b** Venn diagrams showing overlap among DEGs of IVV, IVG, and IVG-NT group, upregulated and downregulated genes, respectively. **c** Four profiles of defined ‘improved genes’ according to the comparison of gene expression level among IVV, IVG, and IVG-NT groups. **d** GO enrichment analysis of ‘improved genes’. **e** Cytoscape plots from ‘improved genes’ and predicted transcription factors (with red circles) were given as an input to be used as potential regulators of the network. **f** KEGG enrichment analysis of ‘improved genes’ which simultaneously targeted by the predicted transcription factors. **g** GO enrichment analysis of ‘improved genes’ which simultaneously targeted by the predicted transcription factors. **h**, **i**, **j** Quantitative RT-PCR detection of *Trp53* (h), *Rac1* (i), *Arf6* (j) transcripts in oocytes from Day 10 follicles. Each group contains three samples from three independent experiments. *, *P* < 0·05. **, *P* < 0·01. ***, *P* < 0·001

As Fig. 4a showed, DEGs were identified through all possible pairwise comparisons among IVV, IVG and IVG-NT groups. Prominent DEGs were found between oocytes from IVV compared to oocytes from IVG. Venn diagrams indicated that 151 upregulated genes and 119 downregulated genes were shared by IVG and IVG-NT group when compared with IVV group separately (Fig. 4b). Eighteen upregulated genes and 14 downregulated genes were shared by IVV and IVG group when compared with IVG-NT group separately.

Genes included in ‘improve’ set were defined as genes showed significantly different expression level between IVV and IVG group, while no significant difference could be found between IVV and IVG-NT group, suggesting the offset effect of NT-4. The set of 139 genes contains four expression patterns, as Fig. 4c represents. GO analysis demonstrated that they got involved in multiple biological processes, such as germ cell development, gamete generation, and cellular process involved in reproduction in multicellular organisms. In addition, biological processes related to meiosis, such as actin filament-based process, actin filament organization, and protein localization to cell periphery, were affected (Fig. 4d). *Trp53*, *Foxa2*, *Stat3,* and *Egr1* were presented as predicted driver transcription factors of the ‘improve’ gene set. When the ‘improve’ gene set and these four transcription factors were included, 67 genes were filtered into the PPI network complex (Fig. 4e). And 26 genes included in a main network where the above transcription factors were served as nodes, were enriched in PI3K-Akt signaling pathway, TGF-beta signaling pathway, Wnt signaling pathway, cell cycle, and cellular senescence using KEGG enrichment analysis (Fig. 4f). Nine GO terms were associated with cell development and proliferation (Fig. 4g). The transcriptional level of key transcription factor (*Trp53*), representative genes of PI3K-Akt signaling pathway (*Rac1*) and actin filament organization (*Arf6)* conformed to the ‘improve’ pattern (Fig. 4h, i and j).

### The effect of NT-4 in oocytes during the IVG process

**Fig. 5:**
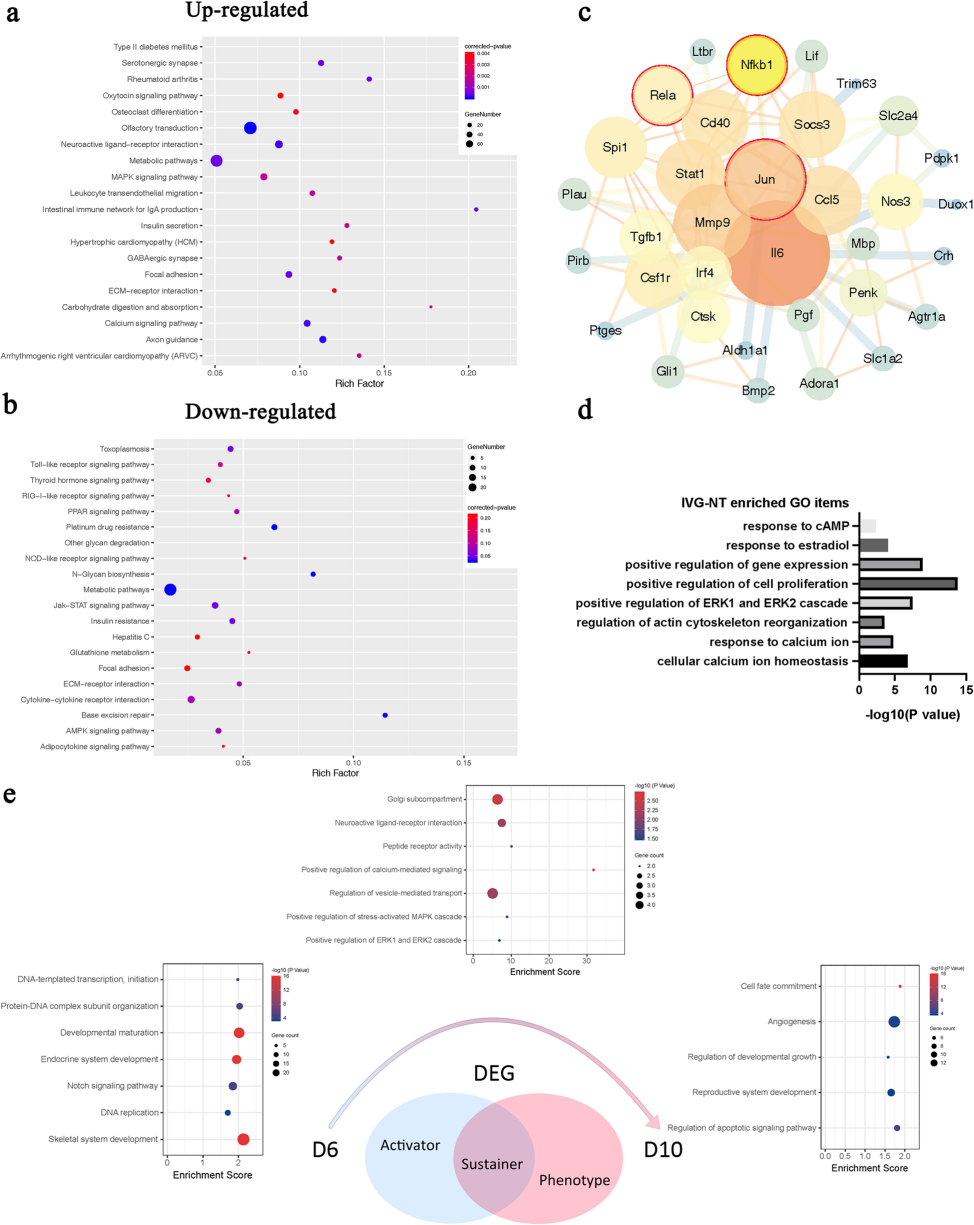
Effect of NT-4 on transcriptome of oocytes from follicles cultured in vitro for six days. **a** KEGG enrichment analysis of upregulated different-expressed genes (DEGs) in IVG-NT oocytes compared with IVG oocytes from follicles cultured in vitro for six days. **b** KEGG enrichment analysis of downregulated DEGs in IVG-NT oocytes than IVG oocytes from follicles cultured in vitro for six days. **c** Cytoscape plots from DEGs and predicted transcription factors (with red circles) were given as an input to be used as potential regulators of the network. **d** GO terms of DEGs which are simultaneously targeted by the predicted transcription factors. **e** Definitions and GO enrichment analyses of ‘Activator’, ‘Sustainer’ and ‘Phenotype’ gene sets according to the overlap of DEGs from oocyte cultured in vitro for ten days (GV) and six days. Each group contains three samples from three independent experiments

We have noticed that the diameter of follicles and level of hormone production had increased with the use of NT-4 since the sixth day of in-vitro culture. Therefore, oocyte and granulosa cells isolated from follicle cultured in vitro for six days were collected as representative for RNA-seq analysis. KEGG analysis of upregulated DEGs (IVG-NT vs. IVG) revealed that genes were enriched in the neuroactive ligand-receptor interaction, MAPK signaling pathway, calcium signaling pathway, and ECM-receptor interaction (Fig. 5a, *P* < 0·01). Corresponding downregulated DEGs were enriched in base excision repair, metabolic pathways, glycan biosynthesis, and degradation pathway (Fig. 5b, *P* < 0·05). *Jun*, *Nfkb1,* and *Rela* were presented as predicted driver transcription factors of DEGs. When the DEGs and predicted transcription factors were included, 34 genes were filtered into the PPI network complex (Fig. 5c), enriched in biological functions related to meiosis, response to estradiol, and positive regulation of cell proliferation (Fig. 5d).

DEGs (IVG-NT vs. IVG) dynamics (from Day 6 to Day 10) of oocytes during IVG were shown in Fig. 5E. ‘Activator’ and ‘phenotype’ gene sets contain genes only differed in Day 6 or Day 10 oocytes respectively, and the overlap of Day 6 and Day 10 DEGs were regarded as ‘sustainer’. The most prominent GO items of ‘activator’ gene set were biological functions related to DNA transcription, DNA replication, development maturation and Notch signaling pathway. DEGs of ‘phenotype’ gene set were enriched in cell fate commitment, angiogenesis, regulation of development growth, reproduction system development and regulation of apoptotic signaling pathway. DEGs sustained throughout the IVG process concentrated on biological functions of golgi subcompartment, neuroactive ligand-receptor interaction, peptide receptor activity, positive regulation of calcium-mediated signaling, regulation of vesicle-mediated transport, positive regulation of stress-activated MAPK cascade and positive regulation of ERK1 and ERK2 cascade.

### The effect of NT-4 in GCs during the IVG process

**Fig. 6:**
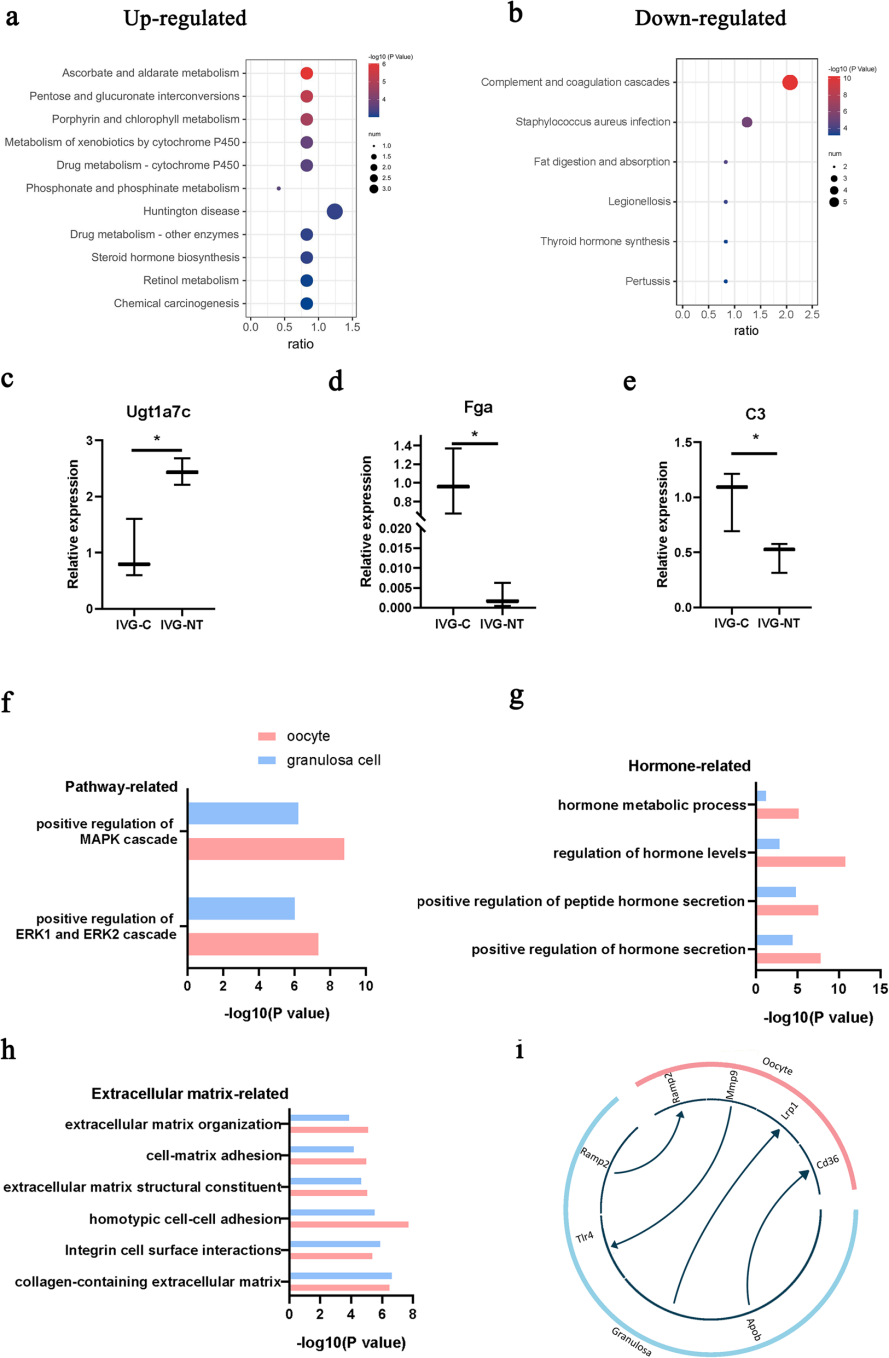
Effect of NT-4 on transcriptome of granulosa cells from Day 6 follicles and their interaction with oocyte. **a** KEGG enrichment of over-expressed genes of granulosa cells in IVG-NT group. **b** KEGG enrichment of over-expressed genes of granulosa cells in IVG group. **c**, **d**, **e** Quantitative RT-PCR detection of *Ugt1a7c* (c), *Fga* (d), *C3* (e) transcripts in granulosa cells from Day 6 follicles. *, *P* < 0·05. **f**, **g**, **h** The overlap of GO enrichment of oocyte and granulosa cells DEGs (IVG-NT vs. IVG), including pathway-related (f), hormone-related (g), and extracellular matrix-related (h). **i** The interaction between oocyte and granulosa cells from follicles cultured in vitro for six days (with or without NT-4) using CellTalkDB tool. Blue represents genes of granulosa cells, and pink represents genes of oocytes. The origin of arrows represents ligand, and the terminal represents receptor. Each group contains two granulosa cell samples from independent experiments

Granulosa cells were isolated from follicles cultured in vitro for six days. Enriching 126 DEGs (IVG-NT vs. IVG), we found that KEGG pathways related to chemical compound metabolism and steroid hormone biosynthesis were upregulated (Fig. 6a), and those getting involved in complement and coagulation cascades, fat digestion, and absorption were downregulated in IVG-NT group (Fig. 6b). The expression of *Fga* and *C3* (representative genes of complement and coagulation cascades pathways) were downregulated, and *Ugt1a7c* (representative genes of steroid hormone biosynthesis) was upregulated in the IVG-NT group than those in the IVG-C group (Fig. 6c, d and e).

### The role of oocyte and granulosa cell communications in NT-4 supplementation

We performed the overlap analysis of GO enrichment of oocyte and granulosa cells DEGs (IVG-NT vs. IVG), and found overlapped GO items were enriched in pathway-related, hormone-related, and extracellular matrix-related biological processes (Fig. 6f, g, h). The Top 4 highly expressed L-R interactions were represented in a circle plot to characterize the L-R–mediated intercellular communication of oocytes and GCs (Fig. 6i).

## Discussion

Our data showed that for mice, TRKB is mainly expressed in oocytes and targeted to GCs as primary follicles initiate growth, in line with previous studies [34]. Limited studies on the human ovary showed that TRKB was immunolocalized to the cytoplasm of oocytes in primordial follicles of human fetal ovaries (18 and 21 wk) [35]. In human adult ovaries [36], immunoreactivity is abundant in oocytes of preovulatory follicles, with lower stains in cumulus cells. Similarly, adult ovaries in our study displayed the location of TRKB in oocytes of primordial/primary and secondary follicles. Using RNA sequencing analysis, NT-4 has been identified as distinct stage-specific and preferentially expressed in human preantral and antral follicles [21]. With gene knock-out mice, a study indicated that the deficiency of TRKB resulted in a diminished number of secondary follicles and FSH receptor expression in the ovary [22]. In addition, a previous study [37] had noted the presence of NT-4 in human follicular fluid samples aspirated from follicles of women undergoing IVF, with a similar level to our data. In this study, we further discovered a positive relationship between oocyte maturation rate and FF NT-4, which, together with the dynamics expression of TRKB and NT-4 in mice and human ovaries, indicated the possible effect of NT-4 on preantral follicle growth.

As expected, we noted an improving effect of NT-4 on follicle growth and oocyte maturation in preantral follicle IVG. The average diameter of follicle and oocyte maturation rate of the control group (without NT-4) were similar to data reported previously [38, 39], revealing the reliability of our IVG system. In detail, a supplement of NT-4 had increased the diameter of follicles since the sixth day of IVG, as well as a significant higher level of steroid hormone production, and finally elevated the maturation rate of GV oocytes at the end of IVG. Collectively, NT-4 had a continuous effect on oocyte and granulosa cells at the preantral and antral follicle stages. Then we collected different cells (oocyte and granulosa cell) from different stages (Day 6 and Day 10) for transcriptome analysis to uncover the underlying mechanism.

Due to the transcription arrest during oocyte maturation, GV oocytes at the end of IVG before IVM were derived to assess the variation NT-4 had led to. Global RNA analysis (heatmap and PCA) of oocytes from follicles in IVV, IVG, and IVG-NT group revealed that NT-4 supplementation to some extent narrow the gap of genes express profile between IVV and IVG. The expression level of typical genes necessary for oogenesis and fully grown oocyte in IVG-NT group also fell in between IVV and IVG group, implicating the qualified biological control of IVG system and compensating effect of NT-4. We further discovered that genes related to neuroactive ligand-receptor interaction were downregulated in oocyte of IVG compared with IVV, supporting our attempt to enhance neuroendocrine regulation in IVG. Consistent with a lower maturation rate of oocytes derived from IVG follicles than IVV follicles, genes related to oocyte maturation and meiosis were also downregulated when cultured in vitro. Upregulated DEGs were highly enriched in pathways related to translation. This supports the notion that fully developed mammalian oocytes become transcriptionally silent prior to meiosis resumption, and standard meiotic is driven mainly by translational utilization of synthesized mRNAs. By translatome profiling, study [40] had found that numbers of transcripts were aberrantly translated in oocytes from aged females compared to young females, indicating that improper translation at the onset of meiosis leads to compromised oocyte quality.

Cross-over analyses with GV oocytes in IVV, IVG, and IVG-NT groups were conducted to understand the improving function of NT-4. Consistent with the notion, more DEGs were found when comparing IVV and IVG groups than groups with or without NT-4. Even so, ‘improve’ genes were found to gather in gamete development and meiosis process, such as actin filament-based process, actin filament organization and protein localization to cell periphery, indicating that the impairment in oocyte maturation of IVG may partly recover with NT-4 supplementation by improvement in actin filament dynamics which has been reported to play important roles during oocyte meiosis [41]. To find the upstream master regulators, driver transcription factors and their protein interaction network were established. One of the driver transcription factors, *Trp53*, is the downstream effector of the neurotrophin signaling pathway activated by NT-4 and TRKB. The function enrichment also indicated that this network referred to PI3K-Akt signaling pathway, cell development, and proliferation. PI3K-Akt signaling pathway has been validated to mediate the organization of F-actin and translocation of the spindle during mouse oocyte meiosis [42]. Collectively, we speculate that NT-4 may upregulate the expression of *Trp53* by neurotrophin signaling pathway, followed by regulation of PI3K-Akt signaling pathway and subsequent organization of F-actin, and thus improve the maturation of GV oocytes.

Oocytes and GCs from preantral follicles on the sixth day of IVG were collected to assess the early effect of NT-4. For oocytes, we found that genes related to calcium signaling and ECM-receptor interaction were upregulated by NT-4 compared with the control group. One of the predicted transcription factors, *Nfkb1*, was also the downstream effector of the neurotrophin signaling pathway. Key regulation network of DEGs referred to pathways essential to oocyte maturation, such as response to cAMP [43], calcium homeostasis [44], actin reorganization [42]. In line with the increasing diameter and hormone production, genes related to response to estradiol and cell proliferation were upregulated by using NT-4. Moreover, establishing stage-specific NT-4 regulation profile, we found that NT-4 mainly regulated the transcription process at the beginning and influenced the cell fate and development growth at the end of IVG. Neurocrine adjustment, calcium-mediated signaling, and vesicle-mediated transport were constantly adjusted by NT-4 during the IVG process, possibly by MAPK cascade or ERK1 and ERK2 cascade.

For GCs, as anticipated, genes upregulated by NT-4 were involved in steroid hormone biosynthesis. Interestingly, complement and coagulation cascades were downregulated by NT-4. Protein in the complement system makes up the major part of mammalian follicular fluid, inducing an aseptic inflammation for follicular rupture and ovulation [45]. However, since granulosa cell samples were collected from the preantral follicles on the sixth day of IVG, advanced stimulation of the complement system and premature induction of the ovulatory process may impair oocyte quality and embryo outcomes, as previously reported [46].

Constructing the cell communication network, we discovered that NT-4 might get involved in the interaction of oocyte and granulosa cells through hormone and extracellular matrix. The effect of NT-4 may be mediated by the connection of ApoB in granulosa cells with CD36 and LRP1 in oocytes. Studies have shown that oocytes from aged mice [47] and obesity females [48] show higher expression of *CD36*, suggesting the elevated lipid uptake. LRP1 is reported to play an essential part in lipid degradation, including ApoB [49]. Collectively, reduced lipid uptake and elevated lipid degradation in IVG-NT oocytes may to some extend recover the inflammation damage induced by abnormal lipid metabolism in IVG environment.

Our research has several limitations. Due to the preciousness of human specimens and irregular distribution of follicles in the human ovary, the detection of TRKB at various stages of folliculogenesis is still inadequate. Though we have collected 15 oocyte samples with different treatments for high-throughput sequencing analyses, the hypotheses about the mechanism require further verification. Owing to the restricted amount of granulosa cells derived from the single-follicle culture system, only two samples of granulosa cells in each group were gathered for bulk RNA-seq analysis. In addition, we hope that with consistent improvement, a preliminary experiment of applying NT-4 in human preantral follicle IVG could be conducted for further clinical application.

To sum up, in this study, we have confirmed the dynamics location of TRKB in human and mice ovaries, as well as a positive relationship between human oocyte maturation and FF NT-4 level, which supports the supplement of NT-4. To the best of our knowledge, this is the first study to find the improving effect of NT-4 on preantral follicle IVG applying mice model, with the goal of developing a more effective and safe method for female fertility preservation. Single-cell RNA sequencing of oocyte combined with cell-specific network analysis (oocyte and granulosa cell) was conducted to uncover the underlying mechanism of effect, which is also the first high-throughput data comparing oocyte derived from preantral follicle IVG versus in vivo. In conclusion, our data suggest that NT-4 is involved in ovarian physiology and may serve as a potentially effective approach to improving the efficiency of preantral follicle IVG for fertility preservation.

## Conclusions

For the first time, we observe the improving effect of NT-4 on preantral follicle IVG applying the mice model, with the goal of developing a more effective and safe method for female fertility preservation. Single-cell RNA sequencing of oocyte combined with cell-specific network analysis (oocyte and granulosa cell) was conducted to uncover the underlying mechanism of effect, which is also the first high-throughput data comparing oocyte derived from preantral follicle IVG versus in vivo. Collectively, our data suggest that NT-4 is involved in ovarian physiology and may serve to improve the efficiency of preantral follicle IVG for fertility preservation.

## Data Availability

Sequence data from this article have been deposited with the DDBJ/EMBL/GenBank Data Libraries under Accession GSE177031 (GSE176516 for oocyte sequence and GSE177030 for granulosa cell sequence).

## Abbreviations

IVG: In-vitro-grow
NT-4: Neurotrophin-4
TRKB: Tropomyosin-related kinase B
FF: Follicular fluid
FP: Fertility preservation
IVF: In vitro fertilization
ICSI: Intracytoplasmic sperm injection
BSA: Bovine serum albumin
COH: Controlled ovarian hyperstimulation
COCs: Cumulus oocyte complexes
GC: Granulosa cell
CCs: Cumulus cells
PCA: Principal Component Analysis
DEGs: Differentially expressed genes
GO: Gene Ontology
KEGG: Kyoto Encyclopedia of Genes and Genomes
GSEA: Gene Set Enrichment Analysis
PPI: Protein–protein network
BMI: Body mass index
AMH: Anti-Müllerian hormone
Gn: Gonadotrophin

## References

[1] Martinez F (2017). Update on fertility preservation from the Barcelona International Society for Fertility Preservation-ESHRE-ASRM 2015 expert meeting: indications, results and future perspectives. Hum Reprod.

[2] Oktay K, Harvey BE, Partridge AH, Quinn GP, Reinecke J, Taylor HS (2018). Fertility preservation in patients with cancer: ASCO clinical practice guideline update. J Clin Oncol.

[3] Yaron Y, Ochshorn Y, Amit A, Kogosowski A, Yovel I, Lessing JB (1998). Oocyte donation in Israel: a study of 1001 initiated treatment cycles. Hum Reprod.

[4] Drakopoulos P, Blockeel C, Stoop D, Camus M, de Vos M, Tournaye H (2016). Conventional ovarian stimulation and single embryo transfer for IVF/ICSI. How many oocytes do we need to maximize cumulative live birth rates after utilization of all fresh and frozen embryos?. Hum Reprod.

[5] Yin H, Kristensen SG, Jiang H, Rasmussen A, Andersen CY (2016). Survival and growth of isolated pre-antral follicles from human ovarian medulla tissue during long-term 3D culture. Hum Reprod.

[6] Horicks F, Van Den Steen G, Gervy C, Clarke HJ, Demeestere I (2018). Both in vivo FSH depletion and follicular exposure to Gonadotrophin-releasing hormone analogues in vitro are not effective to prevent follicular depletion during chemotherapy in mice. Mol Hum Reprod.

[7] Hu Y, Betzendahl I, Cortvrindt R, Smitz J, Eichenlaub-Ritter U (2001). Effects of low O2 and ageing on spindles and chromosomes in mouse oocytes from pre-antral follicle culture. Hum Reprod.

[8] Hardy K, Fenwick M, Mora J, Laird M, Thomson K, Franks S (2017). Onset and heterogeneity of responsiveness to FSH in mouse preantral follicles in culture. Endocrinology.

[9] Thomas FH, Armstrong DG, Telfer EE (2003). Activin promotes oocyte development in ovine preantral follicles in vitro. Reprod Biol Endocrinol.

[10] Kamalamma P, Kona SS, Praveen CV, Siva KA, Punyakumari B, Rao VH (2016). Effect of leptin on in vitro development of ovine preantral ovarian follicles. Theriogenology.

[11] Xu J, Bishop CV, Lawson MS, Park BS, Xu F (2016). Anti-Müllerian hormone promotes pre-antral follicle growth, but inhibits antral follicle maturation and dominant follicle selection in primates. Hum Reprod.

[12] Tkachenko OY, Wolf S, Lawson MS, Ting AY, Rodrigues JK, Xu F (2021). Insulin-like growth factor 2 is produced by antral follicles and promotes preantral follicle development in macaques†. Biol Reprod.

[13] Baba T, Ting AY, Tkachenko O, Xu J, Stouffer RL (2017). Direct actions of androgen, estrogen and anti-Müllerian hormone on primate secondary follicle development in the absence of FSH in vitro. Hum Reprod.

[14] Rodrigues JK, Navarro PA, Zelinski MB, Stouffer RL, Xu J (2015). Direct actions of androgens on the survival, growth and secretion of steroids and anti-Müllerian hormone by individual macaque follicles during three-dimensional culture. Hum Reprod.

[15] Xia X, Wang T, Yin T, Yan L, Yan J, Lu C (2015). Mesenchymal stem cells facilitate in vitro development of human preantral follicle. Reprod Sci.

[16] Aziz A, Fu M, Deng J, Geng C, Luo Y, Lin B, et al. A Microfluidic Device for Culturing an Encapsulated Ovarian Follicle. Micromachines (Basel). 2017;8(11):335. 10.3390/mi8110335 PMC6190016 30400524

[17] Welt CK, Schneyer AL (2001). Differential regulation of inhibin B and inhibin a by follicle-stimulating hormone and local growth factors in human granulosa cells from small antral follicles. J Clin Endocrinol Metab.

[18] McLaughlin M, Albertini DF, Wallace W, Anderson RA, Telfer EE (2018). Metaphase II oocytes from human unilaminar follicles grown in a multi-step culture system. Mol Hum Reprod.

[19] Chang HM, Wu HC, Sun ZG, Lian F, Leung P (2019). Neurotrophins and glial cell line-derived neurotrophic factor in the ovary: physiological and pathophysiological implications. Hum Reprod Update.

[20] Streiter S, Fisch B, Sabbah B, Ao A, Abir R (2016). The importance of neuronal growth factors in the ovary. Mol Hum Reprod.

[21] Zhang Y, Yan Z, Qin Q, Nisenblat V, Chang HM, Yu Y (2018). Transcriptome Landscape of Human Folliculogenesis Reveals Oocyte and Granulosa Cell Interactions. Mol Cell.

[22] Kerr B, Garcia-Rudaz C, Dorfman M, Paredes A, Ojeda SR (2009). NTRK1 and NTRK2 receptors facilitate follicle assembly and early follicular development in the mouse ovary. Reproduction.

[23] Skory RM, Xu Y, Shea LD, Woodruff TK (2015). Engineering the ovarian cycle using in vitro follicle culture. Hum Reprod.

[24] Kim D, Paggi JM, Park C, Bennett C, Salzberg SL (2019). Graph-based genome alignment and genotyping with HISAT2 and HISAT-genotype. Nat Biotechnol.

[25] Kovaka S, Zimin AV, Pertea GM, Razaghi R, Salzberg SL, Pertea M (2019). Transcriptome assembly from long-read RNA-seq alignments with StringTie2. Genome Biol.

[26] Subramanian A, Tamayo P, Mootha VK, Mukherjee S, Ebert BL, Gillette MA (2005). Gene set enrichment analysis: a knowledge-based approach for interpreting genome-wide expression profiles. Proc Natl Acad Sci U S A.

[27] Yu G, Wang LG, Han Y, He QY (2012). clusterProfiler: an R package for comparing biological themes among gene clusters. OMICS.

[28] Han H, Cho JW, Lee S, Yun A, Kim H, Bae D (2018). TRRUST v2: an expanded reference database of human and mouse transcriptional regulatory interactions. Nucleic Acids Res.

[29] Szklarczyk D, Gable AL, Lyon D, Junge A, Wyder S, Huerta-Cepas J (2019). STRING v11: protein-protein association networks with increased coverage, supporting functional discovery in genome-wide experimental datasets. Nucleic Acids Res.

[30] Shannon P, Markiel A, Ozier O, Baliga NS, Wang JT, Ramage D (2003). Cytoscape: a software environment for integrated models of biomolecular interaction networks. Genome Res.

[31] Shao X, Liao J, Li C, Lu X, Cheng J, Fan X. CellTalkDB: a manually curated database of ligand-receptor interactions in humans and mice. Brief Bioinform. 2020;22(4):bbaa269. 10.1093/bib/bbaa269 33147626

[32] Shimamoto S, Nishimura Y, Nagamatsu G, Hamada N, Kita H, Hikabe O (2019). Hypoxia induces the dormant state in oocytes through expression of Foxo3. Proc Natl Acad Sci U S A.

[33] Evsikov AV, Graber JH, Brockman JM, Hampl A, Holbrook AE, Singh P (2006). Cracking the egg: molecular dynamics and evolutionary aspects of the transition from the fully grown oocyte to embryo. Genes Dev.

[34] Paredes A, Romero C, Dissen GA, DeChiara TM, Reichardt L, Cornea A (2004). TrkB receptors are required for follicular growth and oocyte survival in the mammalian ovary. Dev Biol.

[35] Anderson RA, Robinson LL, Brooks J, Spears N (2002). Neurotropins and their receptors are expressed in the human fetal ovary. J Clin Endocrinol Metab.

[36] Seifer DB, Feng B, Shelden RM (2006). Immunocytochemical evidence for the presence and location of the neurotrophin-Trk receptor family in adult human preovulatory ovarian follicles. Am J Obstet Gynecol.

[37] Seifer DB, Feng B, Shelden RM, Chen S, Dreyfus CF (2002). Neurotrophin-4/5 and neurotrophin-3 are present within the human ovarian follicle but appear to have different paracrine/autocrine functions. J Clin Endocrinol Metab.

[38] Lebbe M, Taylor AE, Visser JA, Kirkman-Brown JC, Woodruff TK, Arlt W (2017). The Steroid Metabolome in the Isolated Ovarian Follicle and Its Response to Androgen Exposure and Antagonism. Endocrinology.

[39] Xiao S, Duncan FE, Bai L, Nguyen CT, Shea LD, Woodruff TK (2015). Size-specific follicle selection improves mouse oocyte reproductive outcomes. Reproduction.

[40] Del LE, Masek T, Gahurova L, Pospisek M, Koncicka M, Jindrova A (2020). Age-related differences in the translational landscape of mammalian oocytes. Aging Cell.

[41] Zhang Y, Wu LL, Wan X, Wang HH, Li XH, Pan ZN (2019). Loss of PKC mu function induces cytoskeletal defects in mouse oocyte meiosis. J Cell Physiol.

[42] Zheng P, Baibakov B, Wang XH, Dean J (2013). PtdIns(3,4,5)P3 is constitutively synthesized and required for spindle translocation during meiosis in mouse oocytes. J Cell Sci.

[43] Li HJ, Sutton-McDowall ML, Wang X, Sugimura S, Thompson JG, Gilchrist RB (2016). Extending prematuration with cAMP modulators enhances the cumulus contribution to oocyte antioxidant defence and oocyte quality via gap junctions. Hum Reprod.

[44] Huang N, Yu Y, Qiao J (2017). Dual role for the unfolded protein response in the ovary: adaption and apoptosis. Protein Cell.

[45] Paes VM, Liao SF, Figueiredo JR, Willard ST, Ryan PL, Feugang JM (2019). Proteome changes of porcine follicular fluid during follicle development. J Anim Sci Biotechnol.

[46] Pohler KG, Geary TW, Atkins JA, Perry GA, Jinks EM, Smith MF (2012). Follicular determinants of pregnancy establishment and maintenance. Cell Tissue Res.

[47] Um DE, Shin H, Park D, Ahn JM, Kim J, Song H (2020). Molecular analysis of lipid uptake- and necroptosis-associated factor expression in vitrified-warmed mouse oocytes. Reprod Biol Endocrinol.

[48] Minge CE, Bennett BD, Norman RJ, Robker RL (2008). Peroxisome proliferator-activated receptor-gamma agonist rosiglitazone reverses the adverse effects of diet-induced obesity on oocyte quality. Endocrinology.

[49] Haas ME, Attie AD, Biddinger SB (2013). The regulation of ApoB metabolism by insulin. Trends Endocrinol Metab.

